# The reciprocal relationship between depressive symptoms and deliberate self-harm among Chinese rural adolescents: a cross-lagged panel analysis

**DOI:** 10.3389/fpubh.2024.1422242

**Published:** 2024-11-19

**Authors:** Qijiao Liu, Xiaohe Xu, Jianjun Jiang, Wei Peng, Yuanyi Ji, Ruixi Yang, Ming Zhang, Shiying Li, Yuchen Li, Qiaolan Liu

**Affiliations:** ^1^Department of Health Behavior and Social Medicine, West China School of Public Health and West China Fourth Hospital, Research Center for Palliative Care, West China-PUMC C.C. Chen Institute of Health, Sichuan University, Chengdu, China; ^2^Department of Sociology and Demography, University of Texas at San Antonio, San Antonio, TX, United States; ^3^Department of Palliative Care, West China School of Public Health and West China Fourth Hospital, Research Center for Palliative Care, West China-PUMC C.C. Chen Institute of Health, Sichuan University, Chengdu, China; ^4^Nosocomial Infection Management Department, West China School of Public Health and West China Fourth Hospital, Sichuan University, Chengdu, China; ^5^Department of Sociology and Psychology, School of Public Administration, Sichuan University, Chengdu, China; ^6^Mental Health Center, West China Hospital of Sichuan University, Chengdu, China

**Keywords:** depressive symptom, self-harm, adolescents, mental health, behavioral health, cross-lagged panel model, multi-group analysis

## Abstract

**Background:**

While the association between depressive symptoms and deliberate self-harm in adolescence is extensively documented, the nature, bi-directionality, and longitudinal dynamics of this relationship remain underexplored. This study aims to investigate the causal and reciprocal relationship between depressive symptoms and deliberate self-harm among rural adolescents in western China.

**Methods:**

A 2-year panel study was conducted among 1,840 adolescents aged 10–18 attending rural junior and senior high schools in Sichuan Province, China. The Center for Epidemiologic Studies-Depression Scale (CES-D) and a global measure of self-reported deliberate self-harm were utilized to examine the relationship between depressive symptoms and deliberate self-harm using both classic and random intercept cross-lagged panel models. Multi-group comparisons were carried out for the gender, pubertal stage, and academic performance subgroups.

**Results:**

Positive and statistically significant correlations were found between depressive symptoms and deliberate self-harm both within and across the three waves of the panel survey, after adjusting for covariates, among rural adolescents in western China (*Range*: 0.05–0.28, *p* < 0.05). As anticipated, depressive symptoms positively predicted later deliberate self-harm, which in turn reciprocally predicted subsequent depressive symptoms, both between and within individuals. While the cross-lagged effects were invariant by gender and academic performance, the effect of baseline depressive symptoms on later deliberate self-harm was stronger for adolescents in the early pubertal stage (*β* = 0.19, 95% *Confidence Interval*: 0.08 to 0.30) than for those in the middle-to-late pubertal stage (*β* = 0.13, 95% *CI*: 0.06 to 0.19).

**Conclusion:**

There is a causal and reciprocal relationship between depressive symptoms and deliberate self-harm among rural adolescents in China. Not only does this finding lend further credence to a growing body of research on adolescents’ self-harming behaviors but also informs early intervention strategies aimed at improving behavioral health of rural adolescents in western China.

## 1 Introduction

Depression in adolescence is a common mental health problem. It is often regarded as an early episode of major depression that has a strong link with recurrence in later life (1). Depression can lead to negative and serious behavioral health outcomes, such as substance abuse, deliberate self-harm, or suicidal ideation and attempts (1, 2). In this study, deliberate self-harm refers to intentional self-destruction behavior, irrespective of the type of motive or the extent of suicidal intent (3). For adolescents, self-harm is an expression or regulation of emotional distress such as anxiety and depression (4). It may develop as a symptom of such distress (5). Past research has shown that depression in early adolescence is significantly associated with deliberate self-harm in adulthood (6, 7). A growing body of research has also suggested that self-harm contributes to the increased risk of depression in a reciprocal manner (4, 8, 9). Deliberate self-harm can set the stage for developing depression-like symptoms due to shame or guilt associated with self-harm (9). Additionally, negative reactions from peers and family members toward those exhibiting self-harming behaviors can disrupt interpersonal relationships, further exacerbating depression (10). However, the relationship between depressive symptoms and deliberate self-harm in adolescence remains insufficiently studied. In particular, limited scholarly attention has been given to the nature, bi-directionality, and temporal dynamics of this relationship, especially in the context of rural western China, a socioeconomically underdeveloped region (11). As such, unraveling a possible reciprocal relationship between depressive symptoms and deliberate self-harm in adolescence is essential for uncovering the mechanisms driving this causal process. This knowledge can serve as a crucial step toward developing effective intervention strategies to improve adolescents’ behavioral health in rural China (12).

The causal and reciprocal relationship between depressive symptoms and deliberate self-harm can be best examined by a panel design that measures both variables repeatedly across time. Such repeated measures can be incorporated into a cross-lagged panel model (CLPM) under the structural equation modeling framework (SEM) (13). This type of analysis links the varying degree of depressive symptoms to the change in deliberate self-harm reciprocally over time, assuming a longitudinal relationship between the two variables (14). However, one limitation of the CLPM is that it does not distinguish between between-person and within-person effects (15). To address this, the CLPM can be extended into a random intercept cross-lagged panel model (RI-CLPM), which separates each variable’s variance into between-person variability, which indicates stable characteristics captured by random intercepts, and within-person fluctuations, which represent deviations from an individual’s expected score using novel latent factors (13). Therefore, examining the extent to which within-person effects differ from between-person effects provides a more nuanced understanding of this relationship.

Moreover, to establish a non-spurious relationship between depressive symptoms and deliberate self-harm, a nascent body of research has considered a wide array of factors that potentially confound this relationship. For example, some studies have explored such environmental factors as the parental relationship (16, 17), academic performance (18, 19), and social support (3, 20), all of which are associated with both depressive symptoms and deliberate self-harm, while others have examined individual correlates of depressive symptoms or deliberate self-harm, such as cognitive vulnerability (21), negative responses (22), and negative coping styles (23). Moreover, prior studies have demonstrated that self-esteem (24, 25) and dietary problems (26, 27) are associated with the occurrence of depression or deliberate self-harm, as these factors reflect individual’s psychological states and behavioral patterns.

With regard to sociodemographic characteristics, previous studies have yielded mixed results concerning age and gender differences in depressive symptoms and deliberate self-harm. Although age has been identified as a strong predictor of depressive symptoms in some studies, others found age to be statistically trivial (28). In a similar vein, gender is inconsistently associated with depressive symptoms (28). Turning to self-harming behavior, some studies suggest that girls tend to disproportionately engage in self-harm more than boys (4), whereas other studies failed to replicate this gender difference in the likelihood of engaging in deliberate self-harm (29). Furthermore, the risk of deliberate self-harm increases substantially across the pubertal stage, independent of age (30). Finally, as an important factor for adolescents in school, academic performance has been shown to influence their mental health (31). With these findings in mind, identifying the moderating effects of age, which can be grouped by grades representing different pubertal stages (32), gender, and academic performance on the relationship between depressive symptoms and deliberate self-harm. Identifying these moderating factors not only enhances our understanding of the mechanisms underlying the causal effects but also servers as a practical guide for developing targeted intervention strategies aimed at improving adolescent behavioral health.

The goal of this study is to elucidate the nature and directionality of the relationship between depressive symptoms and deliberate self-harm among rural adolescents in western China. To achieve this goal, we utilize a cross-lagged panel analysis to estimate repeated measures across the three waves of a panel study conducted in rural Sichuan, China, from 2015 to 2017. More specifically, this study tests the following three hypotheses: First, it is hypothesized that there will be longitudinal relationships between depressive symptoms and deliberate self-harm across the three waves of the two-year panel study (H1). Second, it is further surmised that there will be cross-lagged relationships between depressive symptoms and deliberate self-harm across the same study period (H2). Finally, the hypothesized cross-lagged relationships will remain invariant across subgroups defined by gender, pubertal stage, and academic performance (H3).

## 2 Materials and methods

### 2.1 Procedures and participants

A panel study was conducted over a 2-year span from October 2015 to October 2017 in two rural high schools in Zizhong County, Sichuan Province, China. Study participants were junior high students in grades 7–9 (early pubertal stage) and senior high students in grades 10–12 (middle-to-late pubertal stage) (32). A multi-stage sampling technique was employed to select the study subjects. Zizhong County was chosen as our study site because its level of socioeconomic development is representative of that in the province. Two schools (both offering grades 7–12) were randomly selected from the 17 rural high schools in Zizhong County. All junior high students in grade 7 (mean age: 12.2 years, standard deviation, *SD*: 0.7, *Range*: 10–16) and senior high students in grade 10 (mean age: 15.3 years, *SD*: 0.7, *Range*: 12–18) were included as the participants in the baseline survey, which was fielded in October 2015. This survey project featured self-administered questionnaires designed to be completed by the participants. In each survey, participants filled out questionnaires in a classroom setting. To avoid potential learning effects from repeated measures, the sequence of items was altered across surveys. This survey project was approved by the Medical Ethics Committee of Sichuan University (No. 20140307 and Gwll2023117). Written informed consent was secured from all participants and their parents or guardians before the survey was conducted.

This study used data from three survey waves: the baseline survey (October 2015), the second follow-up survey (October 2016), and the fourth follow-up survey (October 2017), in which data on depressive symptoms and deliberate self-harm were available. The original sample sizes from the three surveys were 2,869, 2,750, and 2,457. It is important to note that the number of participants at each survey point fluctuated, as some individuals who participated in the initial survey did not take part in subsequent surveys, while new students joined as participants. Due to sickness, participation in off-campus activities, or transfer, 673 (23.5%) participants from baseline dropped out at the second follow-up (Time 2), and an additional 331 (11.5%) participants continued to drop out at the fourth follow-up (Time 3). Additionally, 25 participants who failed to complete the depression scale and the deliberate self-harm item were excluded. As a result, the analytical sub-sample for the present study comprised 1,840 qualified participants who took part in all three waves. At baseline, of the 1,840 participants, 795 (43.2%) were boys and 1,045 (56.8%) were girls; 384 (20.9%) were junior high students and 1,456 (79.1%) were senior high students, with an average age of 14.6 years (*SD*: 1.4; *Range*: 10–18).

Given the attrition rates of 23.5% at the second follow-up and 11.5% at the fourth follow-up, selection bias was a concern. To test for potential sample selection bias, the basic demographic characteristics (e.g., age, gender, the pubertal stage, and academic performance) and the key variables (i.e., depressive symptoms and deliberate self-harm) were systematically compared between the original baseline sample (*N* = 2,869) and the analytical subsample (*N* = 1,840). Since age (Skewness, *S_k_* = −0.83 < 0; Kurtosis, *K_u_* = −0.04 < 0), depressive symptoms, (*S_k_* = 1.29 > 0; *K_u_* = 1.45 > 0), and deliberate self-harm (*S_k_* = 2.64 > 0; *K_u_* = 6.16 > 0) were not normally distributed in the baseline sample, the rank-sum test was employed. For the two categorical variables—gender and the pubertal stage—the chi-square test was conducted. The rank-sum test results indicated that there were no significant differences in age (*Z* = −1.22, *p* = 0.22 > 0.05), depressive symptoms (*Z* = −1.44, *p* = 0.15 > 0.05), and deliberate self-harm (*Z* = −1.46, *p* = 0.14 > 0.05) between the original baseline sample and the analytical sub-sample. However, the chi-square test results were mixed. While the pubertal stage (*χ^2^*_(1)_ = 0.08, *p* = 0.78 > 0.05) did not significantly differ between the two samples, a gender difference emerged (*χ^2^*_(1)_ = 8.63, *p* = 0.003 < 0.05). That is, more boys were included in the analytical sub-sample than in the original baseline sample. Nevertheless, these results suggest that selection bias is not substantial in this study.

### 2.2 Measures

#### 2.2.1 Depressive symptoms

The Center for Epidemiologic Studies-Depression Scale (CES-D) is a 20-item scale with a total of 60 points designed to measure depressive symptomatology in the general population, including adolescents, with the higher summed scores indicating higher levels of depressive symptoms (33). The Chinese version of the CES-D has been widely used in studies on Chinese adolescents with excellent reliability and validity (34, 35). In the present study, Cronbach’s *α* was 0.930, 0.939, and 0.951 at Time 1, Time 2, and Time 3, respectively.

#### 2.2.2 Deliberate self-harm

Deliberate self-harm came from a global measure that captures the frequency of self-harming behavior in the past year. Participants were asked: “In the past 12 months, how many times have you deliberately harmed yourself, e.g., deliberately cut, burned, scratched, hit body parts, or bit yourself?” The response options included 1 = none, 2 = once, 3 = 2–3 times, and 4 = 4 or more times. Detailed descriptions and assessments can be found elsewhere (4, 36).

#### 2.2.3 Covariates

##### 2.2.3.1 Gender and age

Participants’ self-identified gender and self-reported age were utilized in the present study.

##### 2.2.3.2 Self-esteem

Self-esteem at baseline was measured by the Rosenberg Self-esteem Scale (SES), which contains 10 items (37, 38). All items are rated on a Likert-scale ranging from 1 = strongly disagree to 4 = strongly agree. The sum of the item scores is 40 with higher scores indicating higher levels of self-esteem. Cronbach’s *α* was 0.822.

##### 2.2.3.3 Dietary problems

The following question was used to assess dietary problems at baseline: “Have you had any of the following diet behaviors in the past 30 days?” The response options included 1 = overeating, 2 = abnormally controlling the amount of certain food, 3 = deliberately spitting out the food, 4 = not eating for 24 h or more, 5 = hating to eat vegetables, 6 = hating to eat fruits, 7 = hating to eat meat, and 8 = drinking. The number of adverse dietary behaviors was calculated from the participant’s responses.

##### 2.2.3.4 Parental relationship

One question was used to assess parental relationship at baseline: “How is the relationship between your parents?” The response options included 1 = very good, 2 = good, 3 = medium, 4 = poor, and 5 = very poor.

##### 2.2.3.5 Academic performance

To assess academic performance at baseline, study participants were asked: “How do you think of your academic performance compared with your classmates?” The response options included 1 = poor, 2 = below medium, 3 = medium, 4 = above medium, and 5 = good.

##### 2.2.3.6 Social support

Social support at baseline was measured by the Chinese version of the Social Support Rating Scale (SSRS) (39). The total social support score is the sum of the scores of all items, with higher scores reflecting higher levels of social support. Cronbach’s *α* was 0.722.

### 2.3 Statistical analyses

SPSS 26.0 was utilized for data processing and analysis. The cross-lagged panel models within the structural equation modeling framework were built and estimated using AMOS 26.0. To address the issue of common method variance derived from using the same measurement tool in repeated surveys (40, 41), Harman’s single-factor test was performed before conducting multivariate statistical analyses (42). No significant common method variance surfaced.

Descriptive analyses were performed to report percentages (%) for categorical variables. For non-normally distributed continuous variables, median (*M*) and quartile (*P_25_*, *P_75_*) were reported. As indicated previously, depressive symptoms and deliberate self-harm in the baseline survey were not normally distributed. Thus, the rank-sum tests were employed for independent group comparisons. To streamline statistical analyses, all covariates were collapsed into two or three categories. Age was divided into two categories (< 14 years old and ≥ 14 years old); self-esteem was combined into three categories with ≥30 points = high level of self-esteem, 20–29 points = medium level, and < 20 points = low level of self-esteem (43); the number of the adverse dietary behaviors were dichotomized with 0 = none dietary problems, 1 = mild, and ≥ 2 = noteworthy; parental relationship and academic performance were recoded into three categories (good, medium, and poor), respectively; and social support was also recorded into three categories with low and high categories defined by the 27th and 73rd percentiles (44).

**Figure 1:**
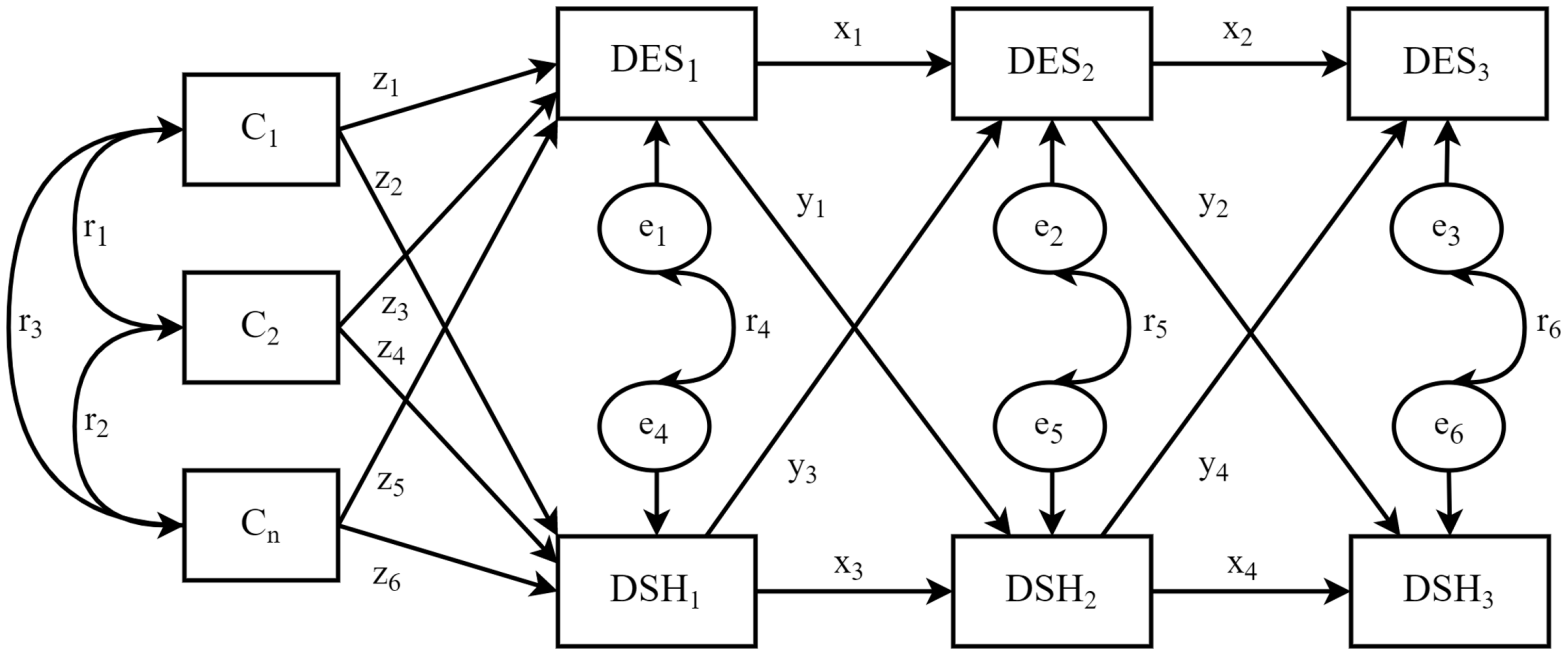
The hypothesized CLPM. DES_1_–DES_3_ represent depressive symptoms at the baseline (October 2015), year one (October 2016), and year two (October 2017), respectively; DSH_1_–DSH_3_ indicate deliberate self-harm at the same points in time; C_1_–C_n_ denote the covariates and e_1_–e_6_ denote the residuals; the parameters x_1_–x_4_ are the autoregressive paths, y_1_–y_4_ are the cross-lagged paths, z_1_–z_6_ are regression paths from covariates predicting depressive symptoms and deliberate self-harm at baseline (Time 1), and r_1_–r_6_ represent correlations between covariates and residual terms.

**Figure 2:**
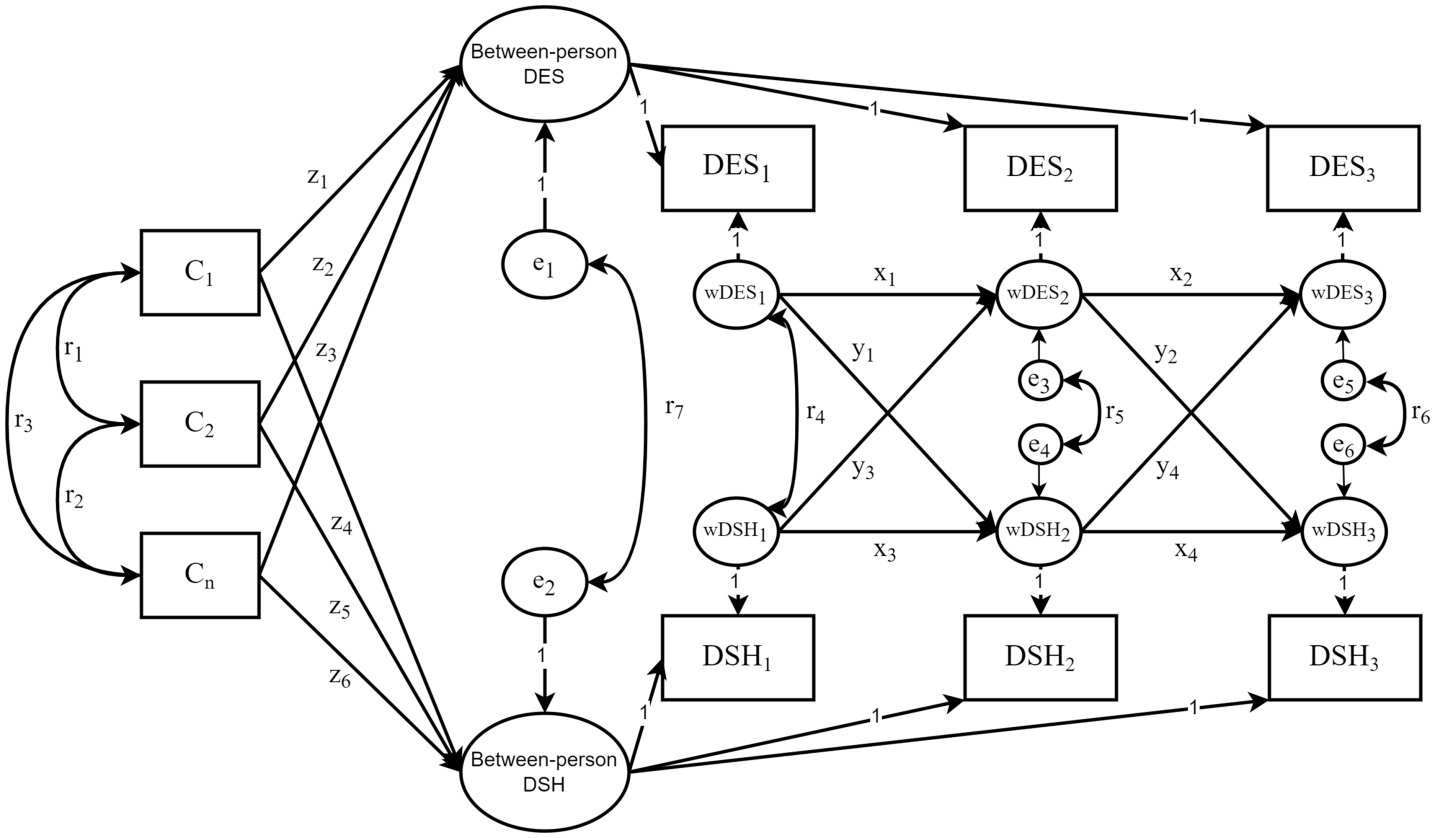
The hypothesized RI-CLPM. “Between-person DES” and “Between-person DSH” represent between-person random intercepts; wDES_1_–wDES_3_ and wDSH_1_–wDSH_3_ indicate within-person random intercepts. All factor loadings were constrained to 1. The other symbols in the diagram share the same meaning as those in Figure 1.

As shown in Figure 1, the CLPM was hypothesized to link depressive symptoms (DES) with deliberate self-harm (DSH). This model features both cross-lagged and autoregressive paths. While the autoregressive parameters (x_1_–x_4_) denote the stability of the variables, the cross-lagged parameters (y_1_–y_4_) indicate causal and reciprocal relationships (13). The standardized path coefficients were used to assess the reciprocal effects of depressive symptoms and deliberate self-harm on each other over time. The covariates (C_1_–C_n_) are the regressors of baseline depressive symptoms (DES_1_) and deliberate self-harm (DSH_1_), and correlations (r_1_–r_6_) between the covariates as well as between the residuals (e_1_–e_6_) are allowed. As shown in Figure 2, the CLPM was extented to the RI-CLPM to assess whether between-person cross-lagged effects could be distinguished from within-person cross-lagged effects (13). The two between-person random intercept latent factors reflect the stable aspects of depressive symptoms and deliberate self-harm over time, while the six within-person random intercept latent factors capture the individuals’ own fluctuating components. The three observed variables of depressive symptoms and deliberate self-harm served as indicators for each latent factor, with all factor loadings set to 1. The error variances of the observed variables were fixed at zero, ensuring that all variation in the observed variables was fully explained by the within-person and between-person latent factors. The invariance of the cross-lagged panel model was also examined using multi-group analysis based on pubertal stage, gender, and academic performance. Furthermore, the bootstrapping approach was utilized to address the issue of non-normal data using 5,000 samples (44). To evaluate the goodness-of-fit of the cross-lagged panel models, the Chi-square statistics divided by the degrees of freedom (*χ^2^*/*df*), the root mean square error of approximation (*RMSEA*), the comparative fit index (*CFI*), the goodness-of-fit index (*GFI*), the Tucker-Lewis index (*TLI*), and the normed fit index (*NFI*) were used and reported. Lower values for *χ^2^*/*df* and *RMSEA*, and higher values for *CFI*, *GFI*, *TLI*, and *NFI* indices indicate better model fit, with *χ^2^*/*df* < 5, *RMSEA* < 0.08, *CFI*, *GFI*, *TLI*, and *NFI* all >0.9 considered as good fit (45). The level of significance was set at 0.05 (two-sided).

## 3 Results

### 3.1 Sample characteristics analyses

Among the 1,840 participants, the median and quartile ranges [*P_25_* and *P_75_*] values of depressive symptoms at the three time points were as follows: 10 [4, 19], 14 [5, 24], and 11 [4, 21]. At these time points, 24.7, 33.5, and 29.9% of individuals exhibited depressive symptoms (defined as a total score of 20 or more), respectively (46). The prevalence of deliberate self-harm (defined as engaging in the behavior from once to four or more times) were 16.3, 10.9, and 10.4%. Additionally, 8.5, 6.3, and 7.1% of participants reported experiencing comorbid depressive symptoms and deliberate self-harm at the corresponding time points. The results of the univariate tests displayed in Table 1 show that the median scores for depressive symptoms were higher for girls than for boys and higher for students in the early pubertal stage than for students in the middle-to-late pubertal stage. Furthermore, adolescents with lower levels of self-esteem, more dietary behavior problems, poorer parental relationships, poorer academic performance, and lower levels of social support exhibited higher levels of depressive symptoms and deliberate self-harm (Bonferroni correction, all *p* < 0.05). However, there were no statistically significant differences in deliberate self-harm across different pubertal stages and age groups.

**Table 1:** Descriptive characteristics of variables at baseline, and univariate tests of depressive symptoms and deliberate self-harm.

	Whole sample	Depressive symptoms	*Z* / *H*	Deliberate self-harm				*Z* / *H*
	*N* (%)	*M* (*P*_25_, *P*_75_)		None (%)	Once (%)	2–3 times (%)	≥ 4 times (%)	
Gender								
Boy	795 (43.2)	8 (3,16)	−5.103***	85.9	6.8	4.3	3.0	−2.185*
Girl	1,045 (56.8)	11 (5,21)		82.0	9.0	5.8	3.2	
Grade (pubertal stage)								
Junior high (early)	384 (20.9)	8 (3,16)	−3.881***	82.6	10.7	3.6	3.1	−0.507
Senior high (middle-to-late)	1,456 (79.1)	11 (4,20)		84.0	7.3	5.7	3.1	
Age								
< 14 years old	391 (21.3)	8 (3,16)	−3.888***	82.4	10.7	3.8	3.1	−0.631
≥ 14 years old	1,449 (78.8)	11 (4,20)		84.1	7.3	5.5	3.1	
Self-esteem								
Low	53 (2.9)	38 (24,51)	208.237***	73.0	11.9	9.5	5.5	46.893***
Medium	1,289 (70.1)	11 (5,20)		88.6	6.2	3.0	2.2	
High	498 (27.1)	6 (2,12)		88.6	6.4	3.4	1.6	
Dietary problems								
None	430 (23.4)	6 (2,15)	95.763***	92.6	4.2	2.8	0.5	57.426***
Mild	731 (39.7)	8 (3,17)		85.6	7.1	4.7	2.6	
Noteworthy	679 (36.9)	13 (6,23)		76.0	11.5	7.2	5.3	
Parental relationship								
Good	1,435 (78.0)	9 (3,17)	66.927***	86.3	7.4	4.0	2.4	33.993***
Medium	311 (16.9)	15 (7,25)		74.9	10.0	9.0	6.1	
Poor	94 (5.1)	15 (6,25)		73.4	11.7	10.6	4.3	
Academic performance								
Poor	690 (37.5)	12 (5,22)	31.088***	79.9	8.8	6.5	4.8	13.345***
Medium	737 (40.1)	10 (4,18)		86.2	8.3	3.5	2.0	
Good	413 (22.4)	7 (3,16)		85.7	6.3	5.8	2.2	
Social support								
Low	498 (27.1)	15 (7,28)	171.152***	76.3	9.0	8.8	5.8	39.471***
Medium	773 (42.0)	10 (4,19)		83.8	9.1	4.5	2.6	
High	569 (30.9)	6 (2,13)		90.0	5.8	2.8	1.4	

The Mann-Whitney U rank sum test whose statistic is *Z* (standardized from *W*) is used for gender, grade (pubertal stage) and age and the Kruskal-Wallis rank sum test whose statistic is *H* is employed for self-esteem, dietary problems, parental relationship, academic performance and social support. *p ≤ 0.05, ***p ≤ 0.001.

### 3.2 Preliminary correlation analysis

The partial correlations between depressive symptoms and deliberate self-harm across the three waves of data collection are reported in Table 2. As shown, all the correlation coefficients were positive and statistically significant after controlling for the covariates (*Range*: 0.05–0.28, all *p* < 0.05), suggesting a consistent longitudinal relationship between depressive symptoms and deliberate self-harm over time. It is interesting to note that the estimated correlations were smaller for the measures of deliberate self-harm (*Range*: 0.15–0.30) than for the measures of depressive symptoms (*Range*: 0.30–0.45) across the three waves. Nevertheless, these results support the first hypothesis (H1).

**Table 2:** Partial correlations between depressive symptoms and deliberate self-harm within and across the three-panel waves.

	Depressive symptoms			Deliberate self-harm		
	Time 1	Time 2	Time 3	Time 1	Time 2	Time 3
Depressive symptoms						
Time 1	1.000					
Time 2	0.38***	1.000				
Time 3	0.30***	0.45***	1.000			
Deliberate self-harm						
Time 1	0.24***	0.15***	0.05*	1.000		
Time 2	0.12***	0.20***	0.16***	0.15***	1.000	
Time 3	0.11***	0.16***	0.28***	0.17***	0.30***	1.000

The partial correlation coefficients are adjusted for gender, age, self-esteem, dietary problems, parental relationship, academic performance, and social support. Time 1–Time 3 denote baseline (October 2015), year one (October 2016), and year two (October 2017) of survey data collection. *p < 0.05, ***p < 0.001.

### 3.3 Cross-lagged panel analyses

#### 3.3.1 Cross-lagged effects in the CLPM

**Figure 3:**
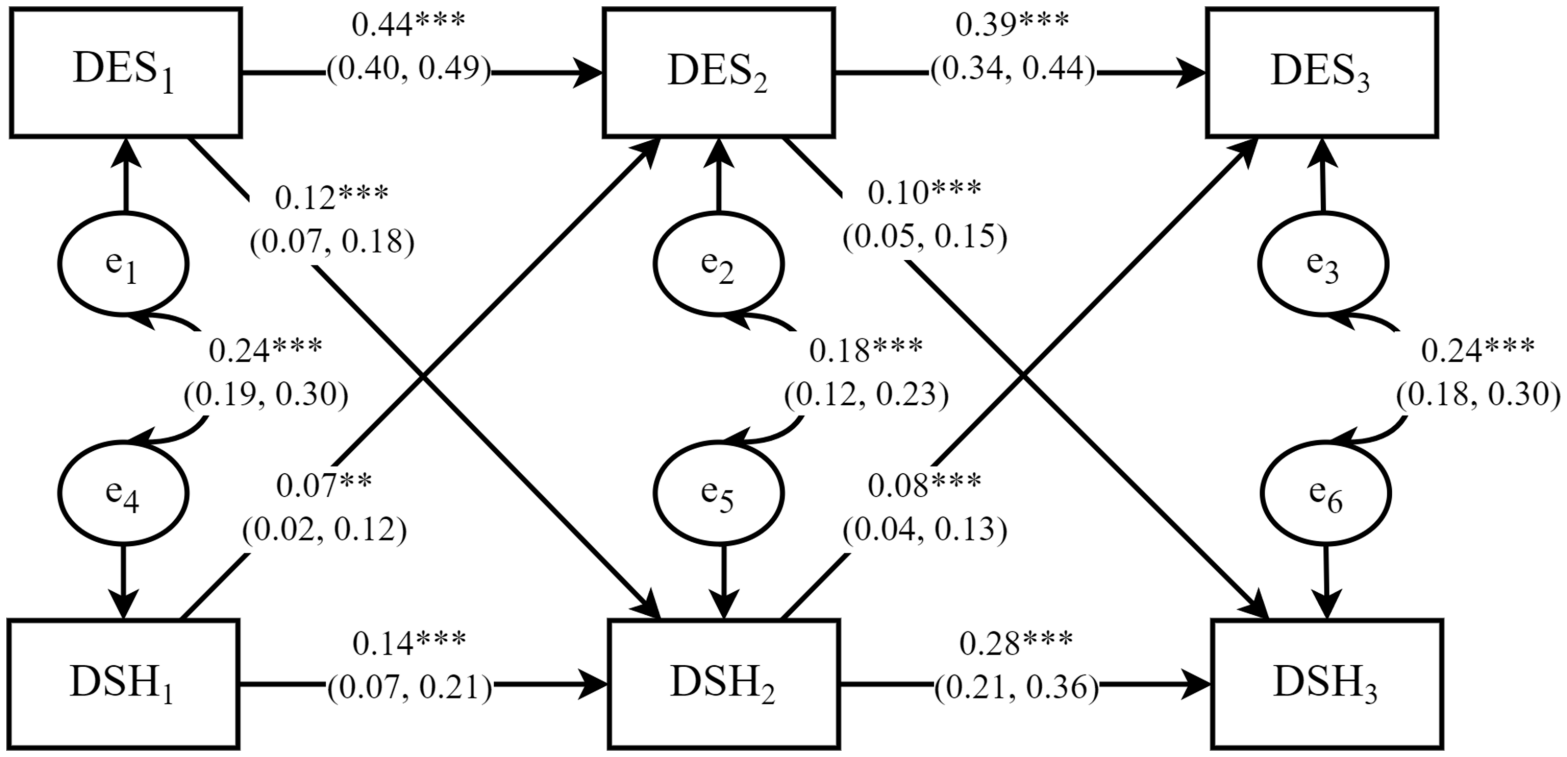
The modified CLPM. Note: The model was adjusted for the seven covariates: gender, age, self-esteem, dietary problems, academic performance, parental relationship, social support. DES_1_-DES_3_ denote depressive symptoms at baseline (October 2015), year one (October 2016), and year two (October 2017) of panel data collection, respectively; DSH_1_–DSH_3_ represent deliberate self-harm at the same points in time, and e_1_–e_6_ denote the residual terms. The solid arrows represent significant causal paths, with the corresponding values indicating standardized path coefficients and 95% confidence interval. **p < 0.01, ***p < 0.001. The parameters for additional paths, as outlined in Figure 1, are reported in Supplementary Table S1 and are not shown in this figure.

Three cross-lagged panel models were estimated. The initial hypothesized model, as depicted in Figure 1, generated an inadequate fit (*TLI* = 0.832). However, after modifying the model to include additional autoregressive paths across the first and third waves (second-order autoregression) (47), the modified CLPM yielded a considerably better model fit, with*χ^2^/df* = 4.860, *RMSEA* = 0.046, *CFI* = 0.964, *GFI* = 0.988, *NFI* = 0.956, and *TLI* = 0.906 (see Figure 3). Importantly, this modification did not change the key results from the hypothesized model (Figure 1). The effect of depressive symptoms at Time 1 on depressive symptoms at Time 3 was statistically significant (*β* = 0.19, 95% *CI:* 0.14 to 0.24), as did the effect of deliberate self-harm at Time 1 on deliberate self-harm at Time 3 (*β =* 0.14, 95% *CI:* 0.08 to 0.20).

As indicated in Table 3 (with unstandardized results) and Figure 3 (with standardized results), all path coefficients were positive and statistically significant in the modified CLPM. There were small but significant concurrent associations between depressive symptoms and deliberate self-harm at each time point, along with small to moderate significant autoregressive paths. A careful examination of the cross-lagged paths reveals that depressive symptoms positively predicted subsequent deliberate self-harm from Time 1 to Time 2 (*β* = 0.12, 95% *CI*: 0.07 to 0.18) and from Time 2 to Time 3 (*β* = 0.10, 95% *CI:* 0.05 to 0.15). These positive cross-lagged effects indicated that the deviations from deliberate self-harm were predicted by depressive symptoms at the previous time point. Moreover, deliberate self-harm positively predicted subsequent depressive symptoms from Time 1 to Time 2 (*β* = 0.07, 95% *CI:* 0.02 to 0.12) and from Time 2 to Time 3 (*β* = 0.08, 95% *CI:* 0.04 to 0.13), confirming positive cross-lagged effects in the opposite direction. The critical ratios (*C.R.*) for differences between parameters indicated statistically significant differences between the effects of depressive symptoms on subsequent deliberate self-harm and the effects of deliberate self-harm on subsequent depressive symptoms from Time 1 to Time 2 (*C.R.* = 3.05 > 2.58, *p* < 0.01) and Time 2 to Time 3 (*C.R.* = −3.94 < −2.58, *p* < 0.01). Stated differently, depressive symptoms and deliberate self-harm positively and reciprocally predicted each other over time, and the causal effects of depressive symptoms on deliberate self-harm were stronger than the reverse effect. Taken together, these findings support the second hypothesis (H2).

**Table 3:** Unstandardized path coefficients for the CLPM and RI-CLPM.

Paths			CLPM				RI-CLPM			
			*B*	*SE*	95% *CI*	*p*-value	*B*	*SE*	95% *CI*	*p*-value
Autoregressive paths										
DES_1_	→	DES_2_	0.47	0.03	(0.42, 0.52)	<0.001	0.17	0.06	(0.05, 0.29)	0.004
DES_2_	→	DES_3_	0.39	0.03	(0.34, 0.44)	<0.001	0.30	0.04	(0.23, 0.37)	<0.001
DSH_1_	→	DSH_2_	0.11	0.03	(0.06, 0.17)	<0.001	−0.03	0.04	(−0.11, 0.05)	0.464
DSH_2_	→	DSH_3_	0.27	0.04	(0.19, 0.35)	<0.001	0.12	0.06	(0.00, 0.24)	0.047
Cross-lagged paths										
DES_1_	→	DSH_2_	0.01	0.00	(0.00, 0.01)	<0.001	0.01	0.00	(0.00, 0.01)	0.098
DES_2_	→	DSH_3_	0.00	0.00	(0.00, 0.01)	<0.001	0.01	0.00	(0.00, 0.01)	0.001
DSH_1_	→	DES_2_	1.23	0.40	(0.33, 2.12)	0.009	1.41	0.57	(0.26, 2.57)	0.018
DSH_2_	→	DES_3_	1.79	0.45	(0.81, 2.80)	<0.001	2.07	0.73	(0.66, 3.53)	0.002

The models were adjusted by the seven covariates: gender, age, self-esteem, dietary problems, academic performance, parental relationship, social support. DES_1_–DES_3_ denote depressive symptoms at baseline (October 2015), year one (October 2016), and year two (October 2017) of survey data collection; DSH_1_–DSH_3_ represent deliberate self-harm at the same points in time; *B* denotes unstandardized path coefficient; *SE* denotes standard error; *CI* represents confidence interval. More coefficient estimates can be found in Supplementary Table S1.

#### 3.3.2 Cross-lagged effects in the RI-CLPM

**Figure 4:**
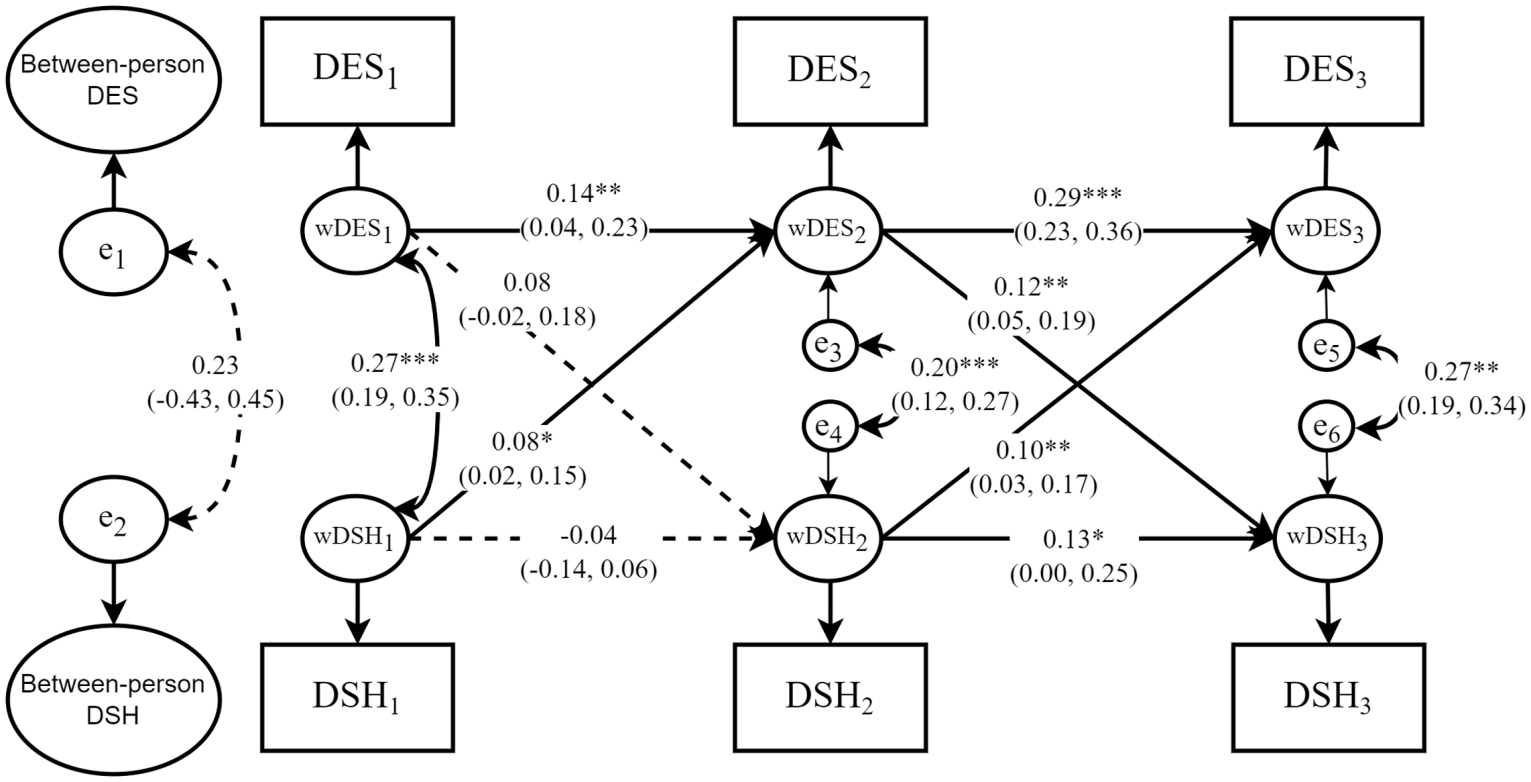
The simplified RI-CLPM. The model was adjusted by the seven covariates: gender, age, self-esteem, dietary problems, academic performance, parental relationship, social support. DES_1_–DES_3_ denote depressive symptoms at baseline (October 2015), year one (October 2016), and year two (October 2017) of panel data collection, respectively; DSH_1_–DSH_3_ represent deliberate self-harm at the same points in time, “Between-person DES” and “Between-person DSH” represent between-person random intercepts; wDES_1_–wDES_3_ and wDSH_1_–wDSH_3_ indicate within-person random intercepts, and e_1_–e_6_ denote the residual terms. The solid arrows represent significant causal paths, with the corresponding values indicating standardized path coefficients and 95% confidence interval. The dashed arrows represent statistically insignificant paths. *p < 0.05, **p < 0.01, ***p < 0.001. The parameters for additional paths, as outlined in Figure 2, are reported in Supplementary Table S1, and are not shown in this figure.

The model fit indices for the RI-CLPM were as follows: *χ^2^/df* = 7.403, *RMSEA* = 0.059, *CFI* = 0.942, *GFI* = 0.983, *NFI* = 0.935, and *TLI* = 0.844, indicating a less optimal fit compared to the CLPM. Figure 4 presents the standardized results, while Table 3 displays the unstandardized results. When comparing the cross-lagged effects between the CLPM and the RI-CLPM, we observed slight differences in the strength of the association between depressive symptoms and deliberate self-harm. The results of the within-person analysis showed significant cross-lagged effects from deliberate self-harm to depressive symptoms. In contrast, the association from depressive symptoms at Time 1 to deliberate self-harm at Time 2 was not significant, though it had an effect size comparable to the significant association in the opposite direction (both *β* = 0.08). However, from Time 2 to Time 3, the RI-CLPM suggested stronger associations from depressive symptoms to deliberate self-harm, consistent with the pattern observed in the CLPM. These findings largely replicate the conclusions of the CLPM, thereby supporting the second hypothesis as well (H2).

Nevertheless, the model fit of the CLPM was superior to that of the RI-CLPM, as indicated by higher *CFI*, and lower *RMSEA* and *SRMR.* This suggests that the more parsimonious CLPM, which assumes a blend of within-person and between-person variances, better aligns with the actual data structure than the RI-CLPM, which separates these effects (48). As such, we should pay more attention to the results from the CLPM.

#### 3.3.3 Cross-lagged effects in subgroups

Based on the CLPM framework, three nested models (M_1_, M_2_, and M_3_) were estimated for subgroups defined by pubertal stage, gender, and academic performance. It is noteworthy that adolescents were categorized into groups based on academic performance as “good” and “poor,” where “good” referred to medium to high levels (original categories 3, 4, and 5), and ‘poor’ referred to levels below medium (original categories 1 and 2). M_1_ was an unconstrained model, M_2_ constrained all path coefficients to be equal, and M_3_ constrained all path coefficients, variances, and covariances to be equal (error variances are rarely constrained to be equal) (49). To carry out the multi-group invariance tests, changes in the model fit indices were utilized as an alternative to the conventional chi-square difference (∆*χ^2^*) that is sensitive to the sample size (50). The absolute value of the change of *CFI* (∆*CFI*) greater than 0.010 indicates that the null hypothesis of multi-group invariance should be rejected (50). For the absolute value of the change of *TLI* (∆*TLI*), the critical value is 0.050 (51).

Since the model fit indices of all unconstrained and constrained models were acceptable (shown in Table 4), they were further tested for the subgroup invariance (47). The test results reported in Table 4 indicate that all the chi-square differences (∆*χ^2^*) between the constrained models and unconstrained models were statistically significant (*p* < 0.05). However, the absolute values of *∆CFIs* and *∆TLIs* were less than their respective critical values across the subgroups of gender, suggesting that the modified cross-lagged panel model was stable across the two gender subgroups. Moreover, nearly all ∆*CFIs* and ∆*TLIs* were less than their respective critical values across the subgroups of the pubertal stage and academic performance except for the ∆*CFI* when M_3_ and M_1_ were compared (∆*CFI* = −0.013 < −0.010), indicating that there were statistically significant differences across the subgroups of the pubertal stage and academic performance. Informed by these test results, the critical ratios (*C.R.*) for differences between parameters across the subgroups of gender, the pubertal stage, and academic performance were generated and examined. As shown in Table 5, there were no statistically significant differences between nearly all the corresponding cross-lagged path coefficients across the subgroups of the pubertal stage, gender, and academic performance except for the path from depressive symptoms at Time 1 to deliberate self-harm at Time 2 across the subgroups of the pubertal stage (*C.R*. = −2.13 < −1.96, *p* < 0.05). The effect of depressive symptoms at Time 1 on deliberate self-harm at Time 2 was stronger in the early pubertal stage (*β* = 0.19, 95% *CI:* 0.08 to 0.30) than in the middle-to-late pubertal stage (*β* = 0.13, 95% *CI:* 0.06 to 0.19). That is, the cross-lagged effects of depressive symptoms and deliberate self-harm were invariant across gender and academic performance, but were different across the pubertal stages, which partially supports the third hypothesis (H3).

**Table 4:** The model fit indices of the unconstrained and constrained models for multi-group analysis and tests for multi-group invariance across the subgroups of pubertal stage, gender, and academic performance.

Groups	Pubertal stage (early and middle-to-late)			Gender (boys and girls)			Academic performance (good and poor)		
Model	M_1_	M_2_	M_3_	M_1_	M_2_	M_3_	M_1_	M_2_	M_3_
χ^2^*/df*	1.939	2.060	1.941	2.996	2.733	2.312	3.213	2.904	2.626
*RMSEA*	0.023	0.024	0.023	0.033	0.031	0.027	0.035	0.032	0.030
*GFI*	0.991	0.987	0.984	0.986	0.982	0.981	0.985	0.981	0.978
*NFI*	0.969	0.953	0.943	0.953	0.938	0.933	0.947	0.932	0.920
*CFI*	0.984	0.975	0.971	0.967	0.959	0.960	0.962	0.953	0.949
*TLI*	0.960	0.955	0.960	0.916	0.927	0.945	0.903	0.916	0.929
*∆CFI*	–	−0.009	−0.013	–	−0.008	−0.007	–	−0.009	−0.013
*∆TLI*	–	−0.005	0.000	–	0.011	0.029	–	0.013	0.026

M_1_ was an unconstrained model, M_2_ constrained all path coefficients to be equal, and M_3_ constrained path coefficients, variances and factor covariances to be equal. The values of *∆CFI* and *∆TLI* were calculated based on the model fit indices of M_1_. For example, the value of *∆CFI* in column for M_2_ was calculated by subtracting the *CFI* of M_1_ from the *CFI* of M_2_.

**Table 5:** Pairwise parameter comparisons across subgroups of pubertal stage, gender, and academic performance in CLPM.

Groups	Cross-lagged paths (Standardized estimates and 95% *CI*)			
	DES_1_ → DSH_2_	DES_2_ → DSH_3_	DSH_1_ → DES_2_	DSH_2_ → DES_3_
Pubertal stage				
Early	0.19 (0.08, 0.30)	0.09 (−0.02, 0.20)	0.04 (−0.06, 0.14)	0.10 (0.00, 0.20)
Middle-to-late	0.13 (0.06, 0.19)	0.10 (0.050, 0.16)	0.07 (0.02, 0.13)	0.04 (−0.00, 0.09)
C.R.	−2.13*	−0.49	0.57	0.72
Gender				
Boys	0.12 (0.03, 0.22)	0.06 (−0.01, 0.13)	0.04 (−0.04, 0.12)	0.06 (−0.01, 0.12)
Girls	0.12 (0.05, 0.19)	0.13 (0.06, 0.19)	0.08 (0.02, 0.15)	0.09 (0.03, 0.15)
C.R.	0.37	1.21	1.01	0.42
Academic performance				
Good	0.15 (0.08, 0.22)	0.10 (0.05, 0.17)	0.03 (−0.03, 0.09)	0.10 (0.04, 0.16)
Poor	0.10 (0.02, 0.19)	0.09 (0.00, 0.17)	0.12 (0.05, 0.20)	0.05 (−0.02, 0.13)
C.R.	−1.34	−0.33	1.91	−0.95

C.R. denotes the critical ratios for differences between parameters. DES_1_–DES_3_ denote depressive symptoms at baseline (October 2015), year one (October 2016), and year two (October 2017) of survey data collection, and DSH_1_–DSH_3_ represent deliberate self-harm at the same time points. *p < 0.05. More coefficient estimates can be found in Supplementary Table S1.

## 4 Discussion

This study investigated the prevalence of depressive symptoms and deliberate self-harm among adolescents in rural western China, with a specific focus on examining the directionality of their relationship. Using the three repeated measures derived from a two-year panel study, our cross-lagged panel analyses demonstrated that there was a causal and reciprocal relationship between depressive symptoms and deliberate self-harm among rural adolescents in Sichuan, China. The average prevalence of depressive symptoms among the adolescents in this study was approximately 30%, which was slightly lower than the reported levels in Asia during the 2010s (40, 95% *CI*: 32–48%) (52). Similarly, the average prevalence of deliberate self-harm was around 13%, lower than the figures reported in Asia during the same period (17.4, 95% *CI*: 12.5–23.7%) (53), with only a modest difference between these estimates. More specifically, there were positive correlations between depressive symptoms and deliberate self-harm over the two-year period. Not only is this result consistent with previous research findings (7, 8) but also supports our first research hypothesis. However, it is worth noting that the correlations between deliberate self-harm at baseline and later depressive symptoms weakened as the interval between measurements increased. These findings make sense because there is a cognitive improvement in adolescents’ perceptions and awareness of the consequences of risky behaviors, such as deliberate self-harm, over time across adolescence (54). Additionally, due to biological and physiological changes, the middle-to-late pubertal stage is a period of time for adolescents to experience difficulties in controlling emotions (4), thus leading to variations in correlational patterns as reported here.

In particular, this study offered robust evidence for the cross-lagged effects concerning the reciprocal relationship between depressive symptoms and deliberate self-harm in adolescence, which lends credence to our second hypothesis. To the best of our knowledge, this is the first study in the Chinese context to examine this bidirectional relationship. Although previous studies have reported unidirectionality between depressive symptoms and deliberate self-harm (4, 6–9), our classic cross-lagged panel analysis revealed that adolescents’ depressive symptoms positively predicted later deliberate self-harm, which in turn, positively predicted subsequent depressive symptoms as well. Notably, the effect of depressive symptoms on self-harm was found to be stronger than the reverse effect. Additionally, the within-person cross-lagged effects observed in the random intercept cross-lagged panel analysis largely replicated these findings. Specifically, changes in individuals’ deviations from expected levels of depressive symptoms were predicted by deviations from their expected levels of deliberate self-harm at the previous time point, with a stronger inverse association observed in the second interval. This reciprocal causal chain is both theoretically and empirically plausible. According to emotion regulation theory, behavior results from emotion regulation processes (55). Adolescents prone to depression often have difficulty in regulating their emotions, so they are more likely to engage in self-injurious behaviors when experiencing negative emotions (56). When adolescents struggle with stressful life events or overwhelmingly adverse emotions, deliberate self-harm can serve as a relatively faster, easier, and more accessible method of relieving themselves, even though it is a maladaptive coping mechanism in dealing with negative emotions (6, 57). Furthermore, adolescents adopting poor emotion regulation are also more likely to exhibit symptoms of psychosis (58). They harm themselves, and tend to be negatively biased when processing information about themselves, thus exhibiting a higher level of psychological distress (21). As such, they are more vulnerable to psychiatric disorders, particularly depression (9). Prior research has empirically supported the emotion regulation theory of non-suicidal self-injury (NSSI) (59), and the findings of this study further extend and enhance this theory to deliberate self-harm.

The multi-group invariance tests yielded three noteworthy results. First, the cross-lagged effects of depressive symptoms and deliberate self-harm were invariant between boys and girls. This is not surprising as prior longitudinal studies have demonstrated that though there are significant gender differences in the onset and acceleration of depressive symptoms and deliberate self-harm in adolescence (4, 16), gender has no bearing on the chronic burden of depressive symptoms and deliberate self-harm (60). Second, subgroup invariance was also found between good and poor academic performers. This result is consistent with previous research findings indicating the weak impact of academic performance on mental health in adolescents (61). Finally, the cross-lagged effects differ significantly across the subgroups of the pubertal stage. That is, the effect of depressive symptoms at baseline on later deliberate self-harm was stronger for the early pubertal stage than for the middle-to-late pubertal stage. This result seems reasonable since junior high adolescents may have poor awareness of the consequences of risky behaviors than their older senior high counterparts (54). Therefore, there is a possibility that the causal relationship between depressive symptoms and later deliberate self-harm is stronger in early adolescence than in middle-to-late adolescence. These findings partially support our third hypothesis.

### 4.1 Implications

The study findings reported above have important practice implications for public health and mental health practitioners. The causal and reciprocal relationship between depressive symptoms and deliberate self-harm among adolescents can form a vicious cycle that may result in suicide (1, 3). However, this reciprocal relationship also implies that the onset of deliberate self-harm could be delayed or controlled if depressive symptoms were managed by early and effective interventions. Similarly, depressive symptoms could be reduced if the risk of deliberate self-harm is minimized. Therefore, intervention programs should target depressive symptoms and deliberate self-harm simultaneously. Particularly, early intervention is important in this regard. Moreover, gender differences were weakened in our study, which suggests that both male and female adolescents may benefit equally from such early intervention programs. Specially, in China, academic performance is often regarded as a factor influencing students’ mental state (31). However, our research results show that students, regardless of academic performance, should be given equal attention with respect to the reciprocal impact. In school settings, developing a sense of school connectedness, rather than focusing on academic performance, may help reduce depressive symptoms and deliberate self-harm among Chinese adolescents (59). Additionally, variables such as self-esteem, parental relationship and social support were included as covariates, and statistically significant regression coefficients were found in our study. As suggested by previous studies, family members who have a poor understanding of depressive symptoms and/or deliberate self-harm during adolescence may inadvertently aggravate poor behavioral health outcomes (10). It is essential for parents, teachers, and other educators to support adolescents in developing accurate self-evaluation skills and learning adaptive strategies for managing negative emotions (62). In summary, early intervention programs should involve collaboration among school, family, and social members who can play a critical and positive role in promoting positive mental health outcomes and supporting adolescents’ rehabilitation process.

### 4.2 Limitations and future directions

Several study limitations must be acknowledged. First, the measurement of deliberate self-harm captured behaviors over past 12 months, while depressive symptoms were assessed for only the preceding week. This discrepancy in timeframes presents a limitation in the study’s design. Given the relatively low frequency of self-harm, a broader measurement window would have been more appropriate, while the CES-D scale for depressive symptoms was necessarily constrained to a one-week period. As a result, caution is warranted when interpreting the longitudinal relationship between these variables. Second, this study utilized a single item to measure deliberate self-harm, which could not distinguish between suicidal self-injury and non-suicidal self-injury. This limits the depth of analysis, and future studies would benefit from employing multi-item research instruments such as the Risk-Taking and Self-Harm Inventory for Adolescents (RTSHIA) (63), the Self-Harm Inventory (SHI) (64), or the Deliberate Self-Harm Inventory (DSHI) (65) to enhance measurement reliability. Third, the coping style (22, 23) and anxiety (66, 67) could potentially confound the relationship between depressive symptoms and deliberate self-harm. Due to the specific research focus of the survey project, these measures were not included in this study but should be considered in future studies. Finally, the causal and reciprocal relationship established in this study via the cross-lagged panel model is based on a multi-wave panel study. Randomized controlled trials can be used in future research to replicate and verify this causal and reciprocal relationship. However, before these sophisticated designs can be implemented in future research, the present study represents a small step forward to document and assess adolescents’ behavioral health in rural China.

## 5 Conclusion

The causal relationship between depressive symptoms and deliberate self-harm is reciprocal after controlling for individual, family, school, and social factors among rural adolescents in western China. This causal and reciprocal relationship remains largely stable within individuals and does not differ significantly by gender or academic performance. However, the relationship is stronger in the early pubertal stage (for junior high adolescents) compared to the later pubertal stage (for senior high adolescents). While these results are in line with the research findings from the West, they offer valuable insights to inform the development of early intervention strategies to address adolescents’ behavioral health needs in the context of rural China.

## Data Availability

The datasets presented in this article are not readily available because the protection of adolescents’ privacy. Requests to access the datasets should be directed to Qiaolan Liu, liuqiaol@scu.edu.cn.

## References

[1] Thapar A Collishaw S Pine DS Thapar AK. Depression in adolescence. Lancet. (2012) 379:1056–67. doi: 10.1016/S0140-6736(11)60871-4, PMID: 22305766 PMC3488279

[2] Plener PL Schumacher TS Munz LM Groschwitz RC. The longitudinal course of non-suicidal self-injury and deliberate self-harm: a systematic review of the literature. Borderline Personal Disord Emot Dysregul. (2015) 2:2. doi: 10.1186/s40479-014-0024-3, PMID: 26401305 PMC4579518

[3] Hawton K Saunders KE O’Connor RC. Self-harm and suicide in adolescents. Lancet. (2012) 379:2373–82. doi: 10.1016/S0140-6736(12)60322-5 22726518

[4] Moran P Coffey C Romaniuk H Olsson C Borschmann R Carlin JB. The natural history of self-harm from adolescence to young adulthood: a population-based cohort study. Lancet. (2012) 379:236–43. doi: 10.1016/S0140-6736(11)61141-0, PMID: 22100201

[5] Lloyd-Richardson EE Perrine N Dierker L Kelley ML. Characteristics and functions of non-suicidal self-injury in a community sample of adolescents. Psychol Med. (2007) 37:1183–92. doi: 10.1017/S003329170700027X, PMID: 17349105 PMC2538378

[6] Rodav O Levy S Hamdan S. Clinical characteristics and functions of non-suicide self-injury in youth. Eur Psychiatry. (2014) 29:503–8. doi: 10.1016/j.eurpsy.2014.02.008, PMID: 24725924

[7] Plener PL Kaess M Schmahl C Pollak S Fegert JM Brown RC. Nonsuicidal self-injury in adolescents. Dtsch Arztebl Int. (2018) 115:23–30. doi: 10.3238/arztebl.2018.0023, PMID: 29366448 PMC5787659

[8] Mars B Heron J Crane C Hawton K Lewis G Macleod J. Clinical and social outcomes of adolescent self harm: population based birth cohort study. BMJ (Clin Res Ed). (2014) 349:349. doi: 10.1136/bmj.g5954, PMID: 25335825 PMC4205277

[9] Wilkinson PO Qiu T Sharon N Jones PB Goodyer IM. Sporadic and recurrent non-suicidal self-injury before age 14 and incident onset of psychiatric disorders by 17 years: prospective cohort study. Br J Psychiatry. (2018) 212:222–6. doi: 10.1192/bjp.2017.45 29514726 PMC7557859

[10] Duarte E Gouveia-Pereira M Gomes HS Sampaio D. How do families represent the functions of deliberate self-harm? A comparison between the social representations from adolescents and their parents. Arch Suicide Res. (2020) 24:173–89. doi: 10.1080/13811118.2018.1545713, PMID: 30537902

[11] Zhou YM Zhao CX Qi YJ He F Huang XN Tian XB. Emotional and behavioral problems of left-behind children in impoverished rural China: a comparative cross-sectional study of fourth-grade children. J Adolesc Health. (2020) 67:S48–54. doi: 10.1016/j.jadohealth.2020.06.016 33246533

[12] Ridley M Rao G Schilbach F Patel V. Poverty, depression, and anxiety: Causal evidence and mechanisms. *Science* (New York, N.Y.). (2020) 370:eaay0214. doi: 10.1126/science.aay0214 33303583

[13] Hamaker EL Kuiper RM Grasman RPPP. A critique of the cross-lagged panel model. Psychol Methods. (2015) 20:102–16. doi: 10.1037/a0038889, PMID: 25822208

[14] López-López JA Kwong ASF Washbrook L Tilling K Fazel MS Pearson RM. Depressive symptoms and academic achievement in UK adolescents: a cross-lagged analysis with genetic covariates. J Affect Disord. (2021) 284:104–13. doi: 10.1016/j.jad.2021.01.091, PMID: 33592428 PMC8105173

[15] Berry D Willoughby MT. On the practical interpretability of cross-lagged panel models: rethinking a developmental workhorse. Child Dev. (2017) 88:1186–206. doi: 10.1111/cdev.12660 27878996

[16] Ma L Gao L Chiu DT Ding Y Wang W Wang Y. Depressive symptoms prevalence, associated family factors, and gender differences: a national cohort study of middle school students in China. J Affect Disord. (2020) 274:545–52. doi: 10.1016/j.jad.2020.05.128, PMID: 32663987

[17] Bifulco A Schimmenti A Moran P Jacobs C Bunn A Rusu AC. Problem parental care and teenage deliberate self-harm in young community adults. Bull Menn Clin. (2014) 78:95–114. doi: 10.1521/bumc.2014.78.2.95 24870845

[18] Liu Y Lu Z. Chinese high school students’ academic stress and depressive symptoms: gender and school climate as moderators. Stress Health. (2012) 28:340–6. doi: 10.1002/smi.2418, PMID: 22190389

[19] Zhang J Song J Wang J. Adolescent self-harm and risk factors. Asia Pac Psychiatry. (2016) 8:287–95. doi: 10.1111/appy.12243 27224048

[20] Gariépy G Helen H Quesnel-Vallée A. Social support and protection from depression: systematic review of current findings in western countries. Br J Psychiatry. (2016) 209:284–93. doi: 10.1192/bjp.bp.115.169094, PMID: 27445355

[21] Beevers CG. Cognitive vulnerability to depression: a dual process model. Clin Psychol Rev. (2005) 25:975–1002. doi: 10.1016/j.cpr.2005.03.003, PMID: 15905008

[22] Nolen-Hoeksema S. Responses to depression and their effects on the duration of depressive episodes. J Abnorm Psychol. (1991) 100:569–82. doi: 10.1037//0021-843x.100.4.569 1757671

[23] Guerreiro DF Cruz D Frasquilho D Santos JC Figueira ML Sampaio D. Association between deliberate self-harm and coping in adolescents: a critical review of the last 10 years’ literature. Arch Suicide Res. (2013) 17:91–105. doi: 10.1080/13811118.2013.776439, PMID: 23614483

[24] Choi Y Choi S Yun J Lim J Kwon Y Young LH. The relationship between levels of self-esteem and the development of depression in young adults with mild depressive symptoms. Medicine (Baltimore). (2019) 98:e17518. doi: 10.1097/MD.0000000000017518, PMID: 31626112 PMC6824750

[25] Junker A Nordahl HM Bjørngaard JH Bjerkeset O. Adolescent personality traits, low self-esteem and self-harm hospitalisation: a 15-year follow-up of the Norwegian Young-HUNT1 cohort. Eur Child Adolesc Psychiatry. (2018) 28:329–39. doi: 10.1007/s00787-018-1197-x 30027416

[26] Khalid S Williams CM Reynolds SA. Is there an association between diet and depression in children and adolescents? A systematic review. Br J Nutr. (2016) 116:2097–108. doi: 10.1017/S0007114516004359 28093091

[27] Berg E Wilhelm K Handley T. Should we increase the focus on diet when considering associations between lifestyle habits and deliberate self-harm? BMC Psychiatry. (2020) 20:560. doi: 10.1186/s12888-020-02950-0, PMID: 33238947 PMC7687696

[28] Bracken BA. Age, race, and gender differences in depressive symptoms: a lifespan developmental investigation. J Psychoeduc Assess. (2010) 28:40–53. doi: 10.1177/0734282909336081

[29] Barrocas AL Giletta M Hankin BL Prinstein MJ Abela JRZ. Nonsuicidal self-injury in adolescence: longitudinal course, trajectories, and intrapersonal predictors. J Abnorm Child Psychol. (2015) 43:369–80. doi: 10.1007/s10802-014-9895-4 24965674

[30] Patton GC Hemphill SA Beyers JM Bond L Toumbourou JW McMORRIS BJ. Pubertal stage and deliberate self-harm in adolescents. J Am Acad Child Adoles Psychiatry. (2007) 46:508–14. doi: 10.1097/chi.0b013e31803065c7, PMID: 17420686

[31] Feng T Jia X Pappas L Zheng X Shao T Sun L. Academic performance and the link with depressive symptoms among rural han and minority Chinese adolescents. Int J Environ Res Public Health. (2022) 19:6026. doi: 10.3390/ijerph19106026, PMID: 35627563 PMC9141636

[32] Chulani VL Gordon LP. Adolescent growth and development. Prim Care. (2014) 41:465–87. doi: 10.1016/j.pop.2014.05.002 25124201

[33] Radloff LS. The CES-D scale: a self-report depression scale for research in the general population. Appl Psychol Meas. (1977) 1:385–401. doi: 10.1177/014662167700100306

[34] Liu J Liu CX Wu T Liu B Jia C Liu X. Prolonged mobile phone use is associated with depressive symptoms in Chinese adolescents. J Affect Disord. (2019) 259:128–34. doi: 10.1016/j.jad.2019.08.017, PMID: 31450134

[35] Chi X Huang L Wang J Zhang P. The prevalence and socio-demographic correlates of depressive symptoms in early adolescents in China: differences in only child and non-only child groups. Int J Environ Res Public Health. (2020) 17:438. doi: 10.3390/ijerph17020438, PMID: 31936468 PMC7014354

[36] Lai S Su C Song S Yan M Tang C Zhang Q. Depression and deliberate self-harm among rural adolescents of Sichuan province in western China: a 2-year longitudinal study. Front Psych. (2021) 12:12. doi: 10.3389/fpsyt.2021.605785 PMC8473622 34589002

[37] Rosenberg M. Conceiving the self. New York: Basic Books (1979).

[38] Tian L. Shortcoming and merits of Chinese version of Rosenberg (1965) self-esteem scale. Psychol Explor. (2006) 2:88–91. doi: 10.3969/j.issn.1003-5184.2006.02.020

[39] Xiao SY. Theoretical foundation and research application about the social support rating scale. J Clin Psychiatry. (1994) 4:98–100.

[40] Campbell DT Fiske DW. Convergent and discriminant validation by the multitrait-multimethod matrix. Psychol Bull. (1959) 56:81–105. doi: 10.1037/h0046016 13634291

[41] Lindell MK Whitney DJ. Accounting for common method variance in cross-sectional research designs. J Appl Psychol. (2001) 86:114–21. doi: 10.1037/0021-9010.86.1.114 11302223

[42] Podsakoff PM Mackenzie SB Lee JY Podsakoff NP. Common method biases in behavioral research: a critical review of the literature and recommended remedies. J Appl Psychol. (2003) 88:879–903. doi: 10.1037/0021-9010.88.5.879, PMID: 14516251

[43] Terra FDS Marziale MHP Robazzi MLDC. Evaluation of self-esteem in nursing teachers at public and private universities. Rev Lat Am Enfermagem. (2013) 21:71–8. doi: 10.1590/s0104-11692013000700010 23459893

[44] Fan F Long K Zhou Y Zheng Y Liu X. Longitudinal trajectories of post-traumatic stress disorder symptoms among adolescents after the Wenchuan earthquake in China. Psychol Med. (2015) 45:2885–96. doi: 10.1017/S0033291715000884, PMID: 25990926

[45] McDonald RP Ho MR. Principles and practice in reporting structural equation analyses. Psychol Methods. (2002) 7:64–82. doi: 10.1037/1082-989x.7.1.64, PMID: 11928891

[46] Garrison CZ Addy CL Jackson KL McKeown RE Waller JL. The CES-D as a screen for depression and other psychiatric disorders in adolescents. J Am Acad Child Adolesc Psychiatry. (1991) 30:636–41. doi: 10.1097/00004583-199107000-00017 1890099

[47] Newsom JT. Longitudinal structural equation modeling: A comprehensive introduction. London: Taylor and Francis (2015).

[48] Masselink M Van Roekel E Hankin BL Keijsers L Lodder G Vanhalst J. The longitudinal association between self-esteem and depressive symptoms in adolescents: separating between-person effects from within-person effects. Eur J Personal. (2018) 32:653–71. doi: 10.1002/per.2179, PMID: 31105382 PMC6519152

[49] Byrne BM. Structural equation modeling with Amos: basic concepts, applications, and programming. 3d ed. London: Taylor and Francis (2016).

[50] Cheung GW Rensvold RB. Evaluating goodness-of-fit indexes for testing measurement invariance. Struct Equ Model Multidiscip J. (2002) 9:233–55. doi: 10.1207/S15328007SEM0902_5

[51] Little TD. Mean and covariance structures (macs) analyses of cross-cultural data: practical and theoretical issues. Multivariate Behav Res. (1997) 32:53–76. doi: 10.1207/s15327906mbr3201_3 26751106

[52] Shorey S Ng ED Wong C. Global prevalence of depression and elevated depressive symptoms among adolescents: a systematic review and meta-analysis. Br J Clin Psychol. (2022) 61:287–305. doi: 10.1111/bjc.12333 34569066

[53] Lim KS Wong CH McIntyre RS Wang J Zhang Z Tran BX. Global lifetime and 12-month prevalence of suicidal behavior, deliberate self-harm and non-suicidal self-injury in children and adolescents between 1989 and 2018: a meta-analysis. Int J Environ Res Public Health. (2019) 16:4581. doi: 10.3390/ijerph16224581 31752375 PMC6888476

[54] Dahl RE. Adolescent brain development: a period of vulnerabilities and opportunities. Ann N Y Acad Sci. (2004) 1021:1–22. doi: 10.1196/annals.1308.001, PMID: 15251869

[55] Gratz KL Roemer L. Multidimensional assessment of emotion regulation and dysregulation: development, factor structure, and initial validation of the difficulties in emotion regulation scale. J Psychopathol Behav Assess. (2004) 26:41–54. doi: 10.1023/B:JOBA.0000007455.08539.94

[56] Dochnal R Vetró Á Kiss E Baji I Lefkovics E Bylsma LM. Emotion regulation among adolescents with pediatric depression as a function of anxiety comorbidity. Front Psych. (2019) 10:722. doi: 10.3389/fpsyt.2019.00722, PMID: 31649566 PMC6790632

[57] Nock MK. Why do people hurt themselves? New insights into the nature and functions of self-injury. Curr Dir Psychol Sci. (2010) 18:78–83. doi: 10.1111/j.1467-8721.2009.01613.x, PMID: 20161092 PMC2744421

[58] Klein RJ Nguyen ND Gyorda JA Jacobson NC. Adolescent emotion regulation and future psychopathology: a prospective transdiagnostic analysis. J Res Adolesc. (2022) 32:1592–611. doi: 10.1111/jora.12743, PMID: 35301763 PMC10152987

[59] Liu S Wu W Zou H Chen Y Xu L Zhang W. Cybervictimization and non-suicidal self-injury among Chinese adolescents: the effect of depression and school connectedness. Front Public Health. (2023) 11:1091959. doi: 10.3389/fpubh.2023.1091959, PMID: 36969626 PMC10030997

[60] Salk RH Petersen JL Abramson LY Hyde JS. The contemporary face of gender differences and similarities in depression throughout adolescence: development and chronicity. J Affect Disord. (2016) 205:28–35. doi: 10.1016/j.jad.2016.03.071, PMID: 27391269 PMC5468750

[61] Agnafors S Barmark M Sydsjö G. Mental health and academic performance: a study on selection and causation effects from childhood to early adulthood. Soc Psychiatry Psychiatr Epidemiol. (2021) 56:857–66. doi: 10.1007/s00127-020-01934-5, PMID: 32813024 PMC8068628

[62] Zou H Chen Z Huo L Kong X Ling C Wu W. The effects of different types of parent-child conflict on non-suicidal self-injury among adolescents: the role of self-criticism and sensation seeking. Curr Psychol. (2024) 43:21019–31. doi: 10.1007/s12144-024-05869-x

[63] Ioanna V Fonagy P Fearon PRM Roussow T. The risk-taking and self-harm inventory for adolescents: development and psychometric evaluation. Psychol Assess. (2010) 22:852–65. doi: 10.1037/a0020583, PMID: 20919771

[64] Sansone RA Wiederman MW. The self-harm inventory (SHI): development of a scale for identifying self-destructive behaviors and borderline personality disorder. J Clin Psychol. (1998) 54:973–83., PMID: 9811134 10.1002/(sici)1097-4679(199811)54:7<973::aid-jclp11>3.0.co;2-h

[65] Gratz KL. Measurement of deliberate self-harm: preliminary data on the deliberate self-harm inventory. J Psychopathol Behav Assess. (2001) 23:253–63. doi: 10.1023/A:1012779403943

[66] Cummings CM Caporino NE Kendal PC. Comorbidity of anxiety and depression in children and adolescents: 20 years after. Psychol Bull. (2014) 140:816–45. doi: 10.1037/a0034733, PMID: 24219155 PMC4006306

[67] Fliege H Lee J Grimm A Klapp BF. Risk factors and correlates of deliberate self-harm behavior: a systematic review. J Psychosom Res. (2008) 66:477–93. doi: 10.1016/j.jpsychores.2008.10.013 19446707

