# Supplementary material for: The reciprocal relationship between depressive symptoms and deliberate self-harm among Chinese rural adolescents: a cross-lagged panel analysis
Source: Front Public Health. 2024 Nov 19;12:1422242. doi: 10.3389/fpubh.2024.1422242 (PMC11611711; doi:10.3389/fpubh.2024.1422242)
Supplement: Supplementary file 1 [file Table_1.DOCX]

Supplementary Material

The reciprocal relationship between depressive symptoms and deliberate self-harm among Chinese rural adolescents: a cross-lagged panel analysis

Qijiao Liu, Xiaohe Xu, Jianjun Jiang, Wei Peng, Yuanyi Ji, Ruixi Yang, Ming Zhang, Shiying Li, Yuchen Li, Qiaolan Liu*

*** Correspondence:** Qiaolan Liu: [liuqiaol@scu.edu.cn](mailto:liuqiaol@scu.edu.cn)

# Supplementary Figures and Tables

## Supplementary Tables

**Supplementary Table 1.** Regression effects for cross-lagged panel models of depressive symptoms and deliberate self-harm.

|  | Paths | *B* | *SE* | *β* (95% *CI*) | *p* |
| --- | --- | --- | --- | --- | --- |
| CLPM | | | | | |
|  | *Causal paths of covariates* |  |  |  |  |
|  | Gender **→** DES_1_ | 1.948 | 0.489 | 0.080 (0.040, 0.118) | 0.001 |
|  | Gender **→** DSH_1_ | 0.051 | 0.032 | 0.036 (-0.009, 0.079) | 0.118 |
|  | Age **→** DES_1_ | 0.343 | 0.169 | 0.041(0.001, 0.079) | 0.043 |
|  | Age **→** DSH_1_ | -0.009 | 0.11 | -0.019 (-0.063, 0.022) | 0.356 |
|  | Self-esteem **→** DES_1_ | -0.855 | 0.060 | -0.320 (-0.369, -0.273) | ＜0.001 |
|  | Self-esteem **→** DSH_1_ | -0.017 | 0.004 | -0.113 (-0.171, -0.058) | ＜0.001 |
|  | Dietary problems **→** DES_1_ | 1.917 | 0.227 | 0.171 (0.131, 0.213) | ＜0.001 |
|  | Dietary problems **→** DSH_1_ | 0.110 | 0.015 | 0.170 (0.122, 0.217) | ＜0.001 |
|  | PR **→** DES_1_ | 0.883 | 0.266 | 0.069 (0.024, 0.113) | 0.001 |
|  | PR **→** DSH_1_ | 0.055 | 0.017 | 0.074 (0.025, 0.124) | 0.004 |
|  | AP **→** DES_1_ | -0.672 | 0.259 | -0.053 (-0.096, -0.011) | 0.013 |
|  | AP **→** DSH_1_ | -0.036 | 0.017 | -0.050 (-0.101, 0.000) | 0.052 |
|  | Social support **→** DES_1_ | -0.374 | 0.048 | -0.171 (-0.216, -0.123) | ＜0.001 |
|  | Social support **→** DSH_1_ | -0.010 | 0.003 | -0.078 (-0.131, -0.024) | 0.004 |
| RI-CLPM | | |  |  |  |
|  | *Causal paths of covariates* |  |  |  |  |
|  | Gender **→** Between DES | 2.134 | 0.401 | 0.142 (-0.088,0.193) | 0.001 |
|  | Gender **→** Between DSH | 0.036 | 0.019 | 0.067 (-0.005,0.136) | 0.068 |
|  | Age **→** Between DES | -0.242 | 0.146 | -0.047 (-0.106,0.008) | 0.088 |
|  | Age **→** Between DSH | -0.044 | 0.008 | -0.237 (-0.330,-0.157) | ＜0.001 |
|  | Self-esteem **→** Between DES | -0.663 | 0.054 | -0.404 (-0.475,-0.338) | ＜0.001 |
|  | Self-esteem **→** Between DSH | -0.014 | 0.003 | -0.230 (-0.335,-0.134) | ＜0.001 |
|  | Dietary problems **→** Between DES | 1.389 | 0.190 | 0.202 (-0.147,0.262) | ＜0.001 |
|  | Dietary problems **→** Between DSH | 0.046 | 0.010 | 0.183 (-0.098,0.272) | 0.001 |
|  | PR **→** Between DES | 0.734 | 0.238 | 0.093 (-0.034,0.155) | 0.002 |
|  | PR **→** Between DSH | 0.028 | 0.011 | 0.097 (-0.017,0.183) | 0.016 |
|  | AP **→** Between DES | -0.541 | 0.227 | -0.070 (-0.129,-0.012) | 0.020 |
|  | AP **→** Between DSH | -0.017 | 0.010 | -0.061 (-0.136,0.010) | 0.091 |
|  | Social support **→** Between DES | -0.315 | 0.045 | -0.235 (-0.298,-0.170) | ＜0.001 |
|  | Social support **→** Between DSH | -0.006 | 0.002 | -0.126 (-0.219,-0.035) | 0.007 |
| CLPM for early pubertal stage | | | | | |
|  | *Autoregressive paths* |  |  |  |  |
|  | DES_1_ **→** DES_2_ | 0.497 | 0.056 | 0.434 (0.326, 0.534) | ＜0.001 |
|  | DES_2_ **→** DES_3_ | 0.423 | 0.059 | 0.373 (0.264, 0.478) | ＜0.001 |
|  | DES_1_ **→** DES_3_ | 0.136 | 0.063 | 0.105 (0.001, 0.212) | 0.049 |
|  | DSH_1_ **→** DSH_2_ | 0.202 | 0.062 | 0.169 (0.018, 0.317) | 0.029 |
|  | DSH_2_ **→** DSH_3_ | 0.362 | 0.049 | 0.370 (0.242, 0.496) | ＜0.001 |
|  | DSH_1_ **→** DSH_3_ | 0.049 | 0.054 | 0.042 (-0.050, 0.143) | 0.363 |
|  | *Cross-lagged paths* |  |  |  |  |
|  | DES_1_ **→** DSH_2_ | 0.013 | 0.004 | 0.188 (0.076, 0.303) | 0.001 |
|  | DES_2_ **→** DSH_3_ | 0.005 | 0.003 | 0.088 (-0.017, 0.195) | 0.094 |
|  | DSH_1_ **→** DES_2_ | 0.717 | 0.945 | 0.037 (-0.060, 0.142) | 0.447 |
|  | DSH_2_ **→** DES_3_ | 1.833 | 0.883 | 0.099 (0.002, 0.201) | 0.046 |
| CLPM for middle-to-late pubertal stage | | | | | |
|  | *Autoregressive paths* |  |  |  |  |
|  | DES_1_ **→** DES_2_ | 0.471 | 0.026 | 0.451 (0.397, 0.504) | ＜0.001 |
|  | DES_2_ **→** DES_3_ | 0.370 | 0.024 | 0.391 (0.336, 0.445) | ＜0.001 |
|  | DES_1_ **→** DES_3_ | 0.232 | 0.024 | 0.234 (0.178, 0.290) | ＜0.001 |
|  | DSH_1_ **→** DSH_2_ | 0.087 | 0.019 | 0.125 (0.051, 0.207) | 0.001 |
|  | DSH_2_ **→** DSH_3_ | 0.172 | 0.024 | 0.183 (0.095, 0.275) | ＜0.001 |
|  | DSH_1_ **→** DSH_3_ | 0.127 | 0.016 | 0.194 (0.116, 0.267) | ＜0.001 |
|  | *Cross-lagged paths* |  |  |  |  |
|  | DES_1_ **→** DSH_2_ | 0.005 | 0.001 | 0.127 0.062, 0.192) | ＜0.001 |
|  | DES_2_ **→** DSH_3_ | 0.004 | 0.001 | 0.104 (0.049, 0.159) | ＜0.001 |
|  | DSH_1_ **→** DES_2_ | 1.309 | 0.442 | 0.072 (0.017, 0.128) | 0.012 |
|  | DSH_2_ **→** DES_3_ | 1.083 | 0.553 | 0.044 (-0.002, 0.088) | 0.060 |
| CLPM for boys | | | | | |
|  | *Autoregressive paths* |  |  |  |  |
|  | DES_1_ **→** DES_2_ | 0.390 | 0.036 | 0.371 (0.295, 0.445) | ＜0.001 |
|  | DES_2_ **→** DES_3_ | 0.332 | 0.035 | 0.326 (0.259, 0.339) | ＜0.001 |
|  | DES_1_ **→** DES_3_ | 0.185 | 0.036 | 0.173 (0.100, 0.250) | ＜0.001 |
|  | DSH_1_ **→** DSH_2_ | 0.084 | 0.028 | 0.110 (0.002, 0.225) | 0.042 |
|  | DSH_2_ **→** DSH_3_ | 0.239 | 0.039 | 0.217 (0.102, 0.340) | 0.001 |
|  | DSH_1_ **→** DSH_3_ | 0.077 | 0.028 | 0.091 (0.015, 0.184) | 0.021 |
|  | *Cross-lagged paths* |  |  |  |  |
|  | DES_1_ **→** DSH_2_ | 0.005 | 0.002 | 0.120 (0.031, 0.216) | 0.008 |
|  | DES_2_ **→** DSH_3_ | 0.003 | 0.002 | 0.060 (-0.010, 0.132) | 0.096 |
|  | DSH_1_ **→** DES_2_ | 0.715 | 0.634 | 0.039 (-0.036, 0.118) | 0.282 |
|  | DSH_2_ **→** DES_3_ | 1.432 | 0.795 | 0.058 (-0.007, 0.122) | 0.080 |
| CLPM for girls | | | | | |
|  | *Autoregressive paths* |  |  |  |  |
|  | DES_1_ **→** DES_2_ | 0.512 | 0.030 | 0.481 (0.420, 0.540) | ＜0.001 |
|  | DES_2_ **→** DES_3_ | 0.423 | 0.029 | 0.429 (0.363, 0.494) | ＜0.001 |
|  | DES_1_ **→** DES_3_ | 0.203 | 0.030 | 0.193 (0.127, 0.256) | ＜0.001 |
|  | DSH_1_ **→** DSH_2_ | 0.133 | 0.028 | 0.153 (0.061, 0.245) | 0.001 |
|  | DSH_2_ **→** DSH_3_ | 0.277 | 0.025 | 0.317 (0.214, 0.419) | ＜0.001 |
|  | DSH_1_ **→** DSH_3_ | 0.129 | 0.021 | 0.171 (0.087, 0.259) | ＜0.001 |
|  | *Cross-lagged paths* |  |  |  |  |
|  | DES_1_ **→** DSH_2_ | 0.006 | 0.002 | 0.120 (0.053, 0.191) | ＜0.001 |
|  | DES_2_ **→** DSH_3_ | 0.005 | 0.001 | 0.125 (0.055, 0.193) | 0.001 |
|  | DSH_1_ **→** DES_2_ | 1.539 | 0.516 | 0.084 (0.023, 0.148) | 0.009 |
|  | DSH_2_ **→** DES_3_ | 1.832 | 0.545 | 0.088 (0.029, 0.151) | 0.003 |
| CLPM for good academic performance | | |  |  |  |
|  | *Autoregressive paths* |  |  |  |  |
|  | DES_1_ **→** DES_2_ | 0.500 | 0.030 | 0.449 (0.390, 0.506) | ＜0.001 |
|  | DES_2_ **→** DES_3_ | 0.361 | 0.029 | 0.361 (0.305, 0.420) | ＜0.001 |
|  | DES_1_ **→** DES_3_ | 0.209 | 0.031 | 0.188 (0.125, 0.252 | ＜0.001 |
|  | DSH_1_ **→** DSH_2_ | 0.145 | 0.027 | 0.158 (0.074, 0.255) | ＜0.001 |
|  | DSH_2_ **→** DSH_3_ | 0.217 | 0.026 | 0.236 (0.138, 0.340) | ＜0.001 |
|  | DSH_1_ **→** DSH_3_ | 0.134 | 0.023 | 0.159 (0.083, 0.242) | ＜0.001 |
|  | *Cross-lagged paths* |  |  |  |  |
|  | DES_1_ **→** DSH_2_ | 0.008 | 0.002 | 0.145 (0.076, 0.217) | ＜0.001 |
|  | DES_2_ **→** DSH_3_ | 0.004 | 0.001 | 0.103 (0.045, 0.166) | ＜0.001 |
|  | DSH_1_ **→** DES_2_ | 0.509 | 0.541 | 0.026 (-0.033, 0.087) | 0.383 |
|  | DSH_2_ **→** DES_3_ | 2.117 | 0.563 | 0.098 (0.041, 0.155) | 0.001 |
| CLPM for poor academic performance | | |  |  |  |
|  | *Autoregressive paths* |  |  |  |  |
|  | DES_1_ **→** DES_2_ | 0.428 | 0.037 | 0.421 (0.339, 0.496) | ＜0.001 |
|  | DES_2_ **→** DES_3_ | 0.424 | 0.036 | 0.427 (0.341, 0.507) | ＜0.001 |
|  | DES_1_ **→** DES_3_ | 0.187 | 0.035 | 0.185 0.106, 0.260) | ＜0.001 |
|  | DSH_1_ **→** DSH_2_ | 0.086 | 0.030 | 0.118 (0.010, 0.230) | 0.032 |
|  | DSH_2_ **→** DSH_3_ | 0.340 | 0.037 | 0.337 (0.211, 0.459) | ＜0.001 |
|  | DSH_1_ **→** DSH_3_ | 0.082 | 0.026 | 0.111 (0.021, 0.207) | 0.017 |
|  | *Cross-lagged paths* |  |  |  |  |
|  | DES_1_ **→** DSH_2_ | 0.004 | 0.002 | 0.100 (0.015, 0.189) | 0.017 |
|  | DES_2_ **→** DSH_3_ | 0.004 | 0.002 | 0.085 (0.004, 0.174) | 0.041 |
|  | DSH_1_ **→** DES_2_ | 2.069 | 0.613 | 0.122 (0.045, 0.201) | 0.003 |
|  | DSH_2_ **→** DES_3_ | 1.221 | 0.758 | 0.053 (-0.017, 0.131) | 0.149 |

Note: The models were adjusted by the seven covariates: gender, age, self-seteem, dietary problems, academic performance, parental relationship, social support. PR is parental relationship, AP is academic performance, DES_1_-DES_3_ denote depressive symptoms at baseline (Oct. 2015), year one (Oct. 2016), and year two (Oct. 2017), and DSH_1_-DSH_3_ represent deliberate self-harm at the same points in time. Between-person DES and Between-person DSH represent the between-person random intercepts. *B* denotes unstandardized path coefficient, *β i*ndicates standardized path coefficient. SE denotes standard error, and *CI* represents confidence interval.
